# Supplementary material for: The First Complete Mitogenome Characterization and Phylogenetic Implications of Elops machnata (Teleostei: Elopiformes: Elopidae)
Source: Biology (Basel). 2025 Jun 21;14(7):739. doi: 10.3390/biology14070739 (PMC12292134; doi:10.3390/biology14070739)
Supplement: Supplementary file 1 [file biology-14-00739-s001.zip › biology-3635971-supplementary.pdf]

Table S1. GenBank accession numbers for Elopomorpha species and outgroups used in constructing the phylogenetic tree.

Class	Order	Family	Organism	Length	Accession No.
Actinopteri	Albuliformes	Albulidae	*Albula glossodonta*	16255	AP002973
			Pterothrissus gissu	16188	NC_005796
			Albula vulpes	16357	NC_082992
	Anguilliformes	Ophichthidae	*Ophichthus erabo*	17856	NC_082578
		Muraenidae	*Gymnothorax odishi*	16579	NC_083140
			Gymnothorax reevesii	16570	PP830653
			Gymnothorax poikilospilus	16572	PQ580705
		Synaphobranchidae	*Synaphobranchus brevidorsalis*	16686	OQ581844
			Synaphobranchus affinis	16677	OQ581845
		Anguillidae	*Anguilla marmorata*	16712	OR475302
	Elopiformes	Elopidae	*Elops machnata* **(this study)**	16712	PV294982
			Elops hawaiensis	16713	NC_005798
			Elops saurus	16120	NC_005803
		Megalopidae	*Megalops cyprinoides*	16412	NC_005799
			Megalops atlanticus	16686	NC_005804
	Notacanthiformes	Notacanthidae	*Notacanthus chemnitzii*	16620	AP018344
			Polyacanthonotus rissoanus	16622	NC_039135
			Notacanthus bonaparte	16623	NC_047186
		Halosauridae	*Halosaurus ovenii*	16647	MW077726
			Aldrovandia affinis	16649	NC_005801
			Halosauropsis macrochir	16655	NC_035885
			Aldrovandia oleosa	16653	NC_039136
			Aldrovandia gracilis	16643	OP056879
	Perciformes	Percidae	*Sander vitreus* **(outgroup)**	16861	OL477730

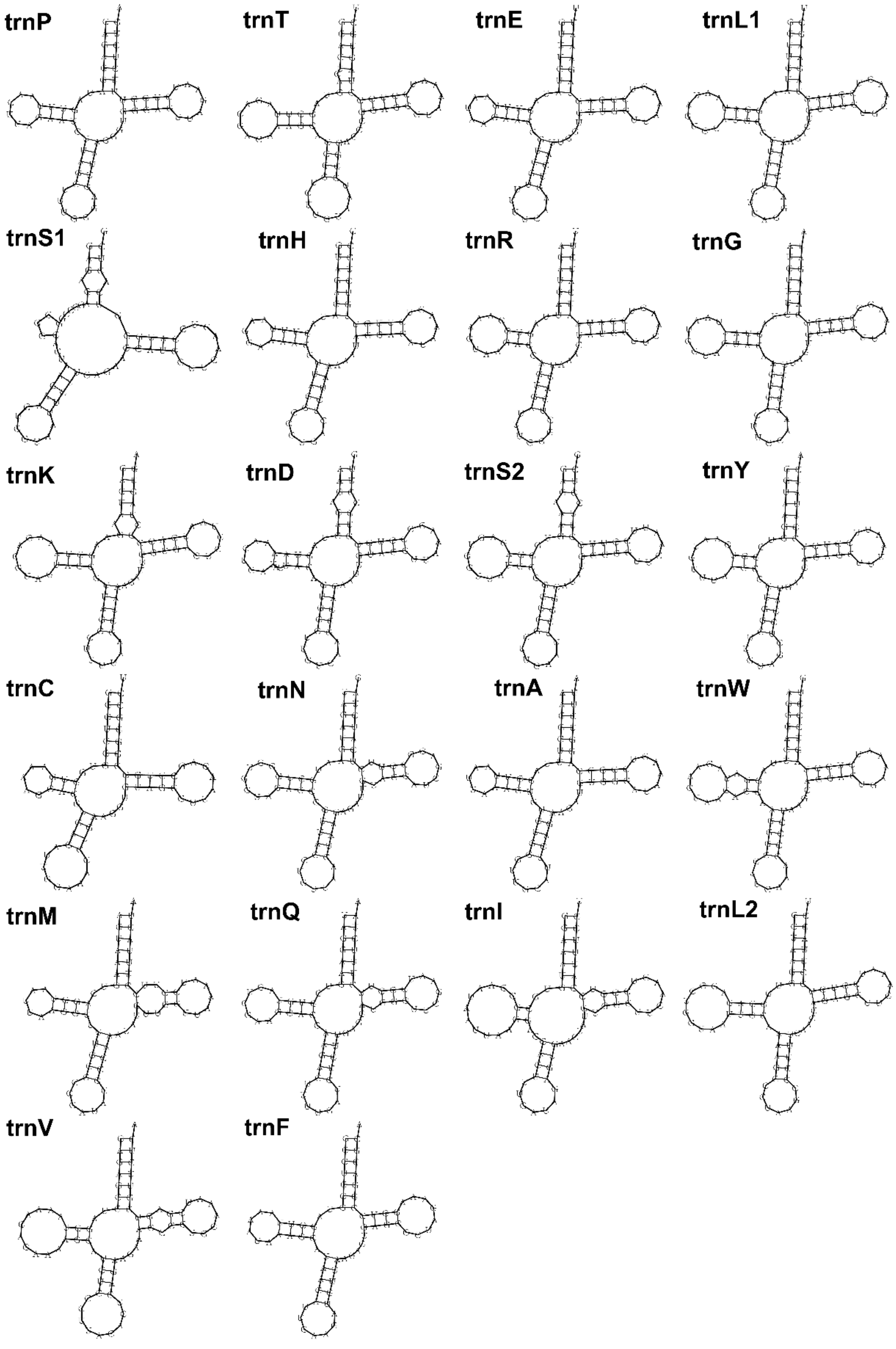

Figure S1. Predicted secondary structures of tRNAs in *E. machnata* mitochondrial genomes.
